# Ste2 receptor-mediated chemotropism of *Fusarium graminearum* contributes to its pathogenicity against wheat

**DOI:** 10.1038/s41598-020-67597-z

**Published:** 2020-07-01

**Authors:** Pooja S. Sridhar, Daria Trofimova, Rajagopal Subramaniam, Dianevys González-Peña Fundora, Nora A. Foroud, John S. Allingham, Michele C. Loewen

**Affiliations:** 1 Department of Biomedical and Molecular Sciences, Queen’s University, 18 Stuart St., Kingston, ON K7L 3N6 Canada; 2 Agriculture and Agri-Food Canada, 960 Carling Avenue, Ottawa, ON K1A 0C6 Canada; 3 Agriculture and Agri-Food Canada, 5403, 1st Avenue South, Lethbridge, AB T1J 4B1 Canada; 4 National Research Council of Canada, 100 Sussex Drive, Ottawa, ON K1A 0R6 Canada

**Keywords:** Fungal pathogenesis, G protein-coupled receptors

## Abstract

Fusarium Head Blight of wheat, caused by the filamentous fungus *Fusarium graminearum,* leads to devastating global food shortages and economic losses. While many studies have addressed the responses of both wheat and *F. graminearum* during their interaction, the possibility of fungal chemotropic sensing enabling pathogenicity remains unexplored. Based on recent findings linking the pheromone-sensing G-protein-coupled receptor Ste2 to host-directed chemotropism in *Fusarium oxysporum*, we investigated the role of the Ste2 receptor and its downstream signaling pathways in mediating chemotropism of *F. graminearum*. Interestingly, a chemotropic response of growing hyphae towards catalytically active *Triticum aestivum* ‘Roblin’ cultivar secreted peroxidases was detected, with deletion of *STE2* in *F. graminearum* leading to loss of the observed response. At the same time, deletion of *STE2* significantly decreased infection on germinating wheat coleoptiles, highlighting an association between Ste2, chemotropism and infection by *F. graminearum*. Further characterization revealed that the peroxidase-directed chemotropism is associated with stimulation of the fungal cell wall integrity mitogen-activated protein kinase signaling cascade. Altogether, this study demonstrates conservation of Ste2-mediated chemotropism by *Fusarium* species, and its important role in mediating pathogenicity.

## Introduction

Filamentous fungi grow by extending their hyphal tips to form an extensive mycelial network, with the hyphal tips often serving as the first point of contact with a new environment. They respond to changes in their environment by directing hyphal growth towards or away from a range of chemical stimuli. Directed hyphal growth towards a chemical stimulus, known as chemotropism, occurs not only in response to nutrient sources and fungal mating factors secreted from opposite mating type cells, but also towards host organisms that the fungi colonize^1–5^. Host-directed chemotropism of fungi is predominantly mediated by G-protein-coupled receptors (GPCRs)^5–7^, and generally leads to spatial proximity of the fungal cells with the host cells, enabling a direct physical interaction and a complex array of molecular responses that underlie the interaction between the two organisms.

GPCRs undergo ligand-mediated conformational changes to transduce extracellular stimuli into intracellular signals. Classically, GPCR stimulation by its ligand leads to the dissociation of its associated heterotrimeric G-protein into α and βγ subunits, which then recruit and activate signalling cascades within the cell, ultimately effecting appropriate biological responses^8^. Originally, it was widely accepted that GPCRs exist exclusively in either an active or inactive conformation, where one GPCR is activated by one ligand resulting in G-protein-mediated signaling and one distinct biological outcome. However, research on G-protein-dependent versus independent signaling of GPCRs over the past two decades has demonstrated that these receptors can exist in multiple conformations depending on the nature of the bound ligand, with different conformations leading to activation of different signaling cascades and biological outcomes. This phenomenon has been termed ‘biased GPCR signaling’ (reviewed extensively^9–11^). While such research has largely been limited to mammalian systems, biased GPCR signaling has been observed in other phyla with the fungal pheromone receptor, Ste2p, in the model organism *Saccharomyces cerevisiae* serving as canonical example^12^.

Ste2p, along with the Ste3p receptor, are pheromone sensing GPCRs in *S. cerevisiae*, encoded by the *STE2* and *STE3* genes. They are expressed on the surface of a- and α-type cells, and recognize α- and a-pheromones secreted by opposite mating type cells, respectively^13–15^. The pheromone receptors and peptides expressed in each cell type are dictated by specific alleles present at the mating-type (*MAT*) locus, *MATα* and *MATa*^16^. Although these loci are present in all fungi, the system is best characterized in *S. cerevisiae*. Classically, binding of α-pheromone peptide to Ste2p activates the pheromone response mitogen-activated protein kinase (MAPK) signalling cascade consisting of Ste11p-Ste7p-Fus1p, leading to cell cycle arrest, shmoo formation and subsequently the formation of a diploid zygote^13^. However, multiple studies have suggested the existence of alternate functionalities for *S. cerevisiae* Ste2p (*Sc*Ste2p)*,* specifically in the mating events that occur downstream of cell cycle arrest, which are influenced by factors such as pheromone gradients^17–20^ as well as localization of the *Sc*Ste2p receptor to the mating projection^21^. Furthermore, specific mutations in ligand-interacting residues of *Sc*Ste2p resulted in different effects on G-protein-mediated MAPK signaling and diploid zygote formation^12,22,23^. In contrast, much less is known about the counterparts of *Sc*Ste2p in multicellular fungi where the mating type of an organism is governed by more complex mechanisms, or where mating is not relevant to the fungal life cycle.

Within the *Fusarium* genus, there exists a diversity of homothallic (fungi that can fertilize themselves to undergo sexual reproduction e.g. *Fusarium graminearum*), heterothallic (fungi that require a compatible partner to undergo sexual reproduction e.g. *Fusarium fujikuroi*) and even asexual (e.g. *Fusarium oxysporum*) species, raising the possibility of diverse roles that both pheromones and their receptors may play in fungal biology. Recently, *F. oxysporum*, a fungal pathogen that causes vascular wilt on many plants including tomatoes^24^, was found to use the Ste2 (*Fo*Ste2) receptor to mediate chemotropism towards the tomato plant roots that it colonizes^5^. This chemotropic growth was shown to be in response to the catalytic product of a tomato root-secreted peroxidase. Rather than activating the pheromone response MAPK signaling pathway, this *Fo*Ste2-mediated response was found to be transduced through the cell wall integrity (CWI) pathway, consisting of *Fo*Bck1-*Fo*Mkk2-*Fo*Mpk1, an alternate MAPK signaling pathway in fungi. Furthermore, both *Fo*Ste2 and *Fo*Ste3 have been shown to regulate conidial germination through autocrine pheromone signaling in *F. oxysporum*^25^. However, whether these alternate functionalities for Ste2 hold true more generally in higher fungi remains to be determined.

*Fusarium graminearum* causes Fusarium Head Blight (FHB) in wheat and other cereal crops, resulting in reduced grain quality and contamination with fungal mycotoxins, leading to severe economic and crop losses worldwide^26^. While the infection biology of the airborne *F. graminearum* has been extensively studied and displays notable differences from that of the soilborne *F. oxysporum*, knowledge regarding *F. graminearum* chemo-sensing and any role it may play in initiation of its infection is lacking.

In addressing this gap, it is important to note that despite its homothallic nature, Ste2 and its respective pheromone remain encoded in the *F. graminearum* genome^27^ (*FgSTE2*). Furthermore, *F. graminearum* also encodes an ortholog to the *F. oxysporum* CWI signalling pathway MAPK protein (*Fg*Mgv1; orthologous to *Fo*Mpk1 in *F. oxysporum*), as well as two other MAPKs, *Fg*Gpmk1 and *Fg*Hog1. Interestingly, while these MAPKs are primarily associated with cell wall integrity and remodelling^28^, pathogenicity and invasion^29,30^, and osmotic stress response^31^, respectively, all three have been implicated in pathogenicity (reviewed by di Pietro et al.^32^). On this basis, wild type and a *STE2* deletion mutant of *F. graminearum* (*Fgste2Δ*) were comparatively tested for their chemotropic responses to a panel of nutrients, metabolites and peroxidases, as well as for their pathogenicity. The *Fg*Ste2 receptor was found to be essential for sensing a wheat peroxidase-derived chemoattractant and its deletion significantly reduced the pathogenicity of *F. graminearum* on germinating wheat coleoptiles. Observed activities were subsequently linked to phosphorylation of the MAPK *Fg*Mgv1, but not *Fg*Gpmk1. Together these findings emphasize the conserved nature of the mechanisms underlying host-mediated chemotropism among *Fusarium* species.

## Results

### Fusarium *graminearum* exhibits chemotropism towards chemical stimuli

A quantitative chemotropism assay^5^ was used to assess the abilities of different compounds to induce directional hyphal growth in *F. graminearum* (Supplemental Figure S1a). Cell concentrations of 0.25 million macroconidia per mL of aqueous agar media were found to be the most suitable for quantifying hyphae. Higher cell concentrations resulted in intertwining hyphae that could not be counted discreetly, while concentrations lower than 0.25 million per mL yielded insufficient numbers of cells on the scoring line. Growth of conidia exposed to gradients of various compounds was monitored microscopically and an optimum period of 14 h was chosen for counting hyphae. For consistency, only conidia with single hypha were included; those with more than one germinating hypha were excluded from the count.

**Figure 1:**
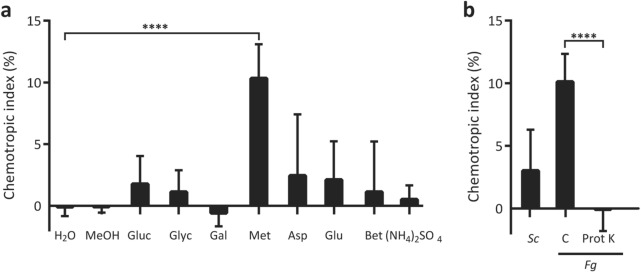
Wild type *F. graminearum* exhibits chemotropic growth towards nutrients and fungal pheromones. (**a**) Directed hyphal growth of wild type *F. graminearum* towards a gradient of specified nutrient sources after 14 h exposure; *MeOH* methanol, *Gluc* glucose, *Glyc* glycerol, *Gal* galactose, *Met* methionine, *Asp* aspartate, *Glu* glutamate, *Bet* betaine, *(NH*_*4*_*)*_*2*_*SO*_*4*_ ammonium sulfate (compared to solvent control, ****P < 0.0001). (**b**) Directed hyphal growth of *F. graminearum* towards a gradient of α-pheromone of *S. cerevisiae* (*Sc*) and *F. graminearum* (*Fg*), either untreated (C) or treated with Proteinase K (Prot K) (compared to untreated control, ****P < 0.0001). Data represent the average from at least three experiments. n = 500 hyphae. Error bars represent standard deviation. Graphs were plotted using Graphpad Prism version 6.01 (https://www.graphpad.com). The figure was compiled using Adobe Illustrator CC 2015 (https://www.adobe.com).

Nutrients with nitrogen and/or carbon sources were screened first for their ability to induce chemotropism in wild type *F. graminearum* (Fig. 1a). The chemotropic responses to nutrients were compared with a double-negative control plate where water was added to both the test and control wells. Among nutrients, a significant chemotropic response was induced by methionine where ~ 10% more macroconidia grew hyphae towards methionine compared to the water control (Fig. 1a, Supplemental Figure S1b). Responses towards other nutrients were highly variable, and none of them elicited a response as robust or significant as methionine. Interestingly, it was observed that exposure of wild type macroconidia to any of the nitrogen-containing compounds, including glutamate, aspartate, ammonium sulfate and betaine, caused rapid hyphal growth yielding long, intertwined hyphae, compared to exposure to other nutrients. Nonetheless, with the exception of methionine, this rapid growth was not found to be significantly directional and thus was not deemed chemotropic in its nature.

With *Fg*Ste2 being an α-pheromone receptor, it was expected that exposure of *F. graminearum* to the α-pheromone would stimulate signaling through the receptor and ultimately a chemotropic response towards it. Hence, chemically synthesized α-pheromone peptides of *S. cerevisiae* and *F. graminearum* were next screened against wild type *F. graminearum* macroconidia (Fig. 1b). The two α-pheromone peptides are of similar size, but differ in sequence and thus, specificity. Exposure to *Fg* α-pheromone (Supplemental Figure S1c) stimulated a robust chemotropic response compared to the double-negative water control, while *Sc* α-pheromone induced a weaker and more variable response. To validate that it is the α-pheromone peptide that elicits directional growth, the *F. graminearum* α-pheromone was proteolyzed with proteinase K, resulting in a complete loss of chemotropism (Fig. 1b).

### *Fusarium graminearum* exhibits positive chemotropism toward the wheat head and secreted wheat peroxidases

**Figure 2:**
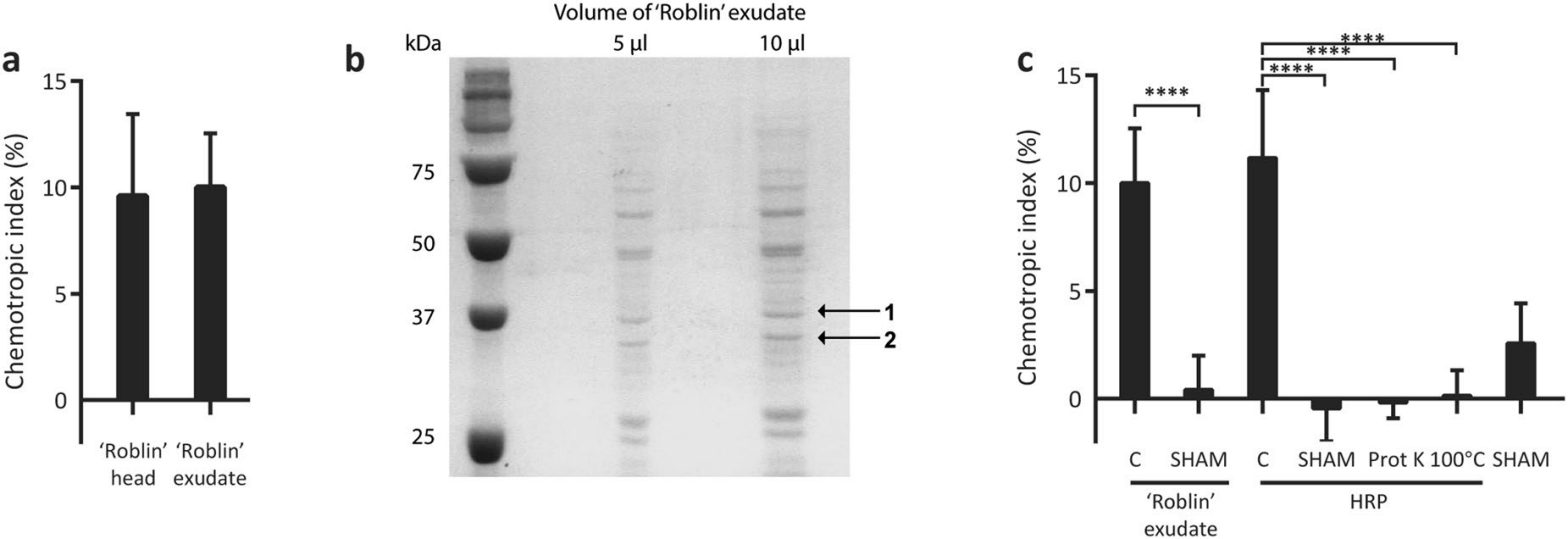
Wild type *F. graminearum* exhibits chemotropic growth in response to wheat head-secreted peroxidases. (**a**) Directed hyphal growth of *F. graminearum* towards the ‘Roblin’ wheat head and 300 × concentrated ‘Roblin’ exudate. (**b**) SDS-PAGE of ‘Roblin’ exudate followed by staining with Coomassie blue. Protein bands excised and identified by mass spectrometry are labelled 1 and 2. Molecular weight markers are indicated on the left. (**c**) Directed hyphal growth of *F. graminearum* towards ‘Roblin’ exudate, either untreated (C) or inhibited with salicylhydroxamic acid (SHAM) and horseradish peroxidase (HRP) untreated (C), inhibited with SHAM, proteolyzed by proteinase K (Prot K) or boiled at 100 °C (compared to untreated control, ****P < 0.0001). Data represents the average of at least three experiments. n = 500 hyphae. Error bars represent standard deviation. Graphs were plotted using Graphpad Prism version 6.01 (https://www.graphpad.com). The figure was compiled and labelled using Adobe Illustrator CC 2015 (https://www.adobe.com).

Macroconidia of *F. graminearum* are dispersed onto the wheat head prior to initiation of infection, implying that any wheat-derived chemoattractant inducing chemotropism would likely originate from the wheat head. This provided a rationale for investigating the wheat head alone in inducing directional hyphal growth in wild type *F. graminearum*. Preliminary chemotropism assays using wheat heads of cultivars having different susceptibilities towards *F. graminearum*, including the highly susceptible ‘Roblin’, moderately resistant ‘Wuhan’, and highly resistant ‘Sumai3’ cultivars, were conducted (Supplemental Figure S2a). While each of the wheat heads elicited chemotropic responses in wild type *F. graminearum*, these preliminary studies do not suggest any correlation between susceptibility and the intensity of the chemotropic response (Supplemental Figure S2b). Experiments were subsequently limited to the most susceptible cultivar ‘Roblin’. To facilitate further investigation into the nature of the host molecules that induce this response, exudate from the spikelets of ‘Roblin’ was extracted and tested for chemotropic effect. The response to ‘Roblin’ exudate (Supplemental Figure S1d) was similar to that seen for the intact ‘Roblin’ wheat head (Fig. 2a), confirming that the ‘chemoattractant’ is likely a water-soluble molecule derived from the wheat head. A concentration-dependent chemotropic response was seen towards ‘Roblin’ exudate, with a more concentrated exudate inducing a stronger chemotropic response in *F. graminearum* (Supplemental Figure S2c).

Toward identification of potential chemoattractant proteins, ‘Roblin’ exudate was analyzed by SDS-PAGE (Fig. 2b). Mass spectrometric peptide fingerprinting of two bands (labeled 1 and 2) identified a variety of proteins including four wheat peroxidases, with NCBI protein IDs SPT21090, CDM85516, SPT21091, and SPT16353, corresponding to the expected molecular weights of these accessions, 35.5, 36.8, 38.2 and 41.0 kDa, respectively (Supplementary Figure S3a). Moreover, transcriptomic analysis of wheat infected with *F. graminearum* (NCBI SRA sample BioSample accession SAMN04386757)^33^ shows an upregulation of three of these four wheat peroxidases; SPT21090 (fourfold, padj = 2.07 × 10^–9^), CDM85516 (2.5-fold, padj = 6.15 × 10^–3^), and SPT21091 (fourfold, padj = 2.07 × 10^–9^). To confirm that the detected secreted peroxidases are functional, catalytic activity of the ‘Roblin’ exudate was tested against a well-known peroxidase substrate, pyrogallol, in the presence of hydrogen peroxide, and compared with commercially available horse radish peroxidase (HRP) (Supplemental Figure S3b, S3c). The ‘Roblin’ exudate exhibited robust catalytic activity in the pyrogallol assay.

The role of one or more of these peroxidases in eliciting chemoattraction was further examined by treatment of the ‘Roblin’ exudate with the peroxidase-specific inhibitor salicylhydroxamic acid (SHAM), prior to assessing chemotropism (Fig. 2c). The observed elimination of any chemotropic response toward the SHAM-inhibited ‘Roblin’ exudate by *F. graminearum* validates the importance of active peroxidases secreted by the wheat head in this system.

While wheat peroxidases are valid candidates to contribute to the stimulation of chemotropism, HRP was tested in the chemotropism assay as a more reliable and simplified assay system. Conservation of essential catalytic residues between HRP and the identified wheat peroxidases provides a rationale for similar chemotropic responses of *F. graminearum* towards the two stimuli (Supplemental Figure S3a). As expected, exposure to HRP induced a robust chemotropic response in wild type *F. graminearum* (Fig. 2c, Supplemental Figure S1e). This HRP-induced chemotropism was completely abolished either by proteinase K treatment or by boiling at 100 °C. Inhibition of HRP with SHAM also eliminated the chemotropic response. These findings emphasize that the chemoattractant is not the peroxidase itself, but the product of a peroxidase-catalyzed reaction.

**Figure 3:**
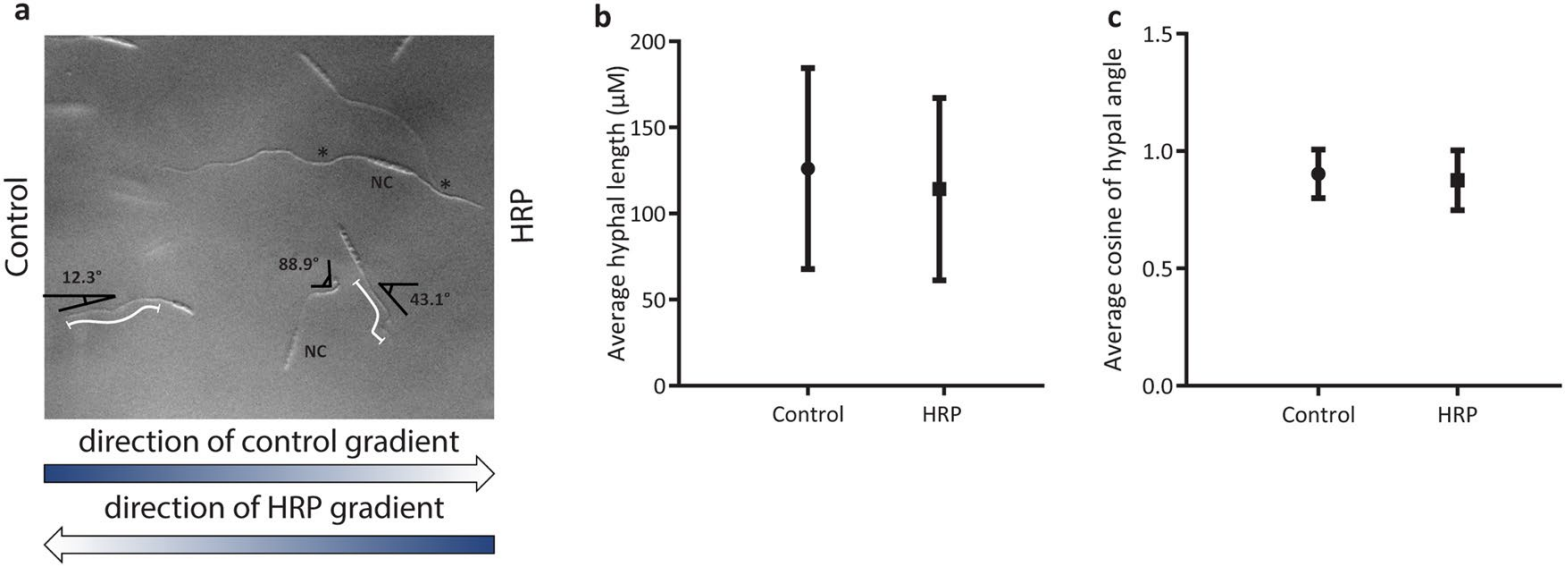
Response of wild type *F. graminearum* towards HRP in chemotropism plate assay is not due to growth speed or subjective bias. (**a**) Representative image of length measurements and angles of counted hyphae growing towards HRP and water control. Direction of gradients of HRP and control compounds are indicated on the image. Examples of hyphal length measurements are depicted by the white lines along the hyphae. Hyphae marked NC were not counted because of large angle (> 45°) with respect to the gradient and multiple hyphae germinating from one conidia (marked with asterisk). Image contrast has been increased for clarity Using ImageJ (https://imagej.nih.gov/ij/). (**b**) Average length of hyphae of wild type *F. graminearum* towards a gradient of HRP or water control. Data is representative of two experiments. n = 300 hyphae. Error bars represent standard deviation. (**c**) Average cosine of hyphal angle of *F. graminearum* towards a gradient of HRP or water control with respect to the direction of the respective gradient. Data is representative of two experiments. n = 300 hyphae. Error bars represent standard deviation. Graphs were plotted using Graphpad Prism version 6.01 (https://www.graphpad.com). The figure was compiled and labelled using Adobe Illustrator CC 2015 (https://www.adobe.com).

Finally, to confirm that this observed response was indeed chemotropism and not a growth speed or subjective bias, the lengths and angles of the hyphae growing towards test (HRP) and control (water) compounds in the chemotropism plate assay were measured (n = 300, Fig. 3a–c). This analysis showed no significant differences in the lengths, and thus no growth speed bias, of hyphae growing towards HRP compared to those growing towards the water control (Fig. 3b). To avoid subjective bias, only hyphae with angles of 0 to approximately 45° were counted and used for calculation of the chemotropic index. The angles of hyphae that were counted as growing towards the HRP and water control were measured (Fig. 3c) and a similar distribution of angles was observed for both HRP and the water control. No significant difference was observed between the cosine of angles of these hyphae with the average cosine of 0.87, corresponding to an angle of approximately 28° (Fig. 3c).

### Deletion of *F. graminearum STE2* results in loss of chemotropic response

To investigate the role of *Fg*Ste2 in chemotropism, the previously annotated *STE2* gene^27,34,35^ was deleted through homologous recombination using ATMT. Three positive hygromycin-resistant transformants (*Fgste2Δ-1*, *Fgste2Δ-3*, *Fgste2Δ-5*) were further confirmed by PCR amplification across the upstream junction of integration of the knockout cassette with primers P13 (located in the upstream genomic DNA region outside of the knockout cassette) + P16 (within the hygromycin coding region) (Supplemental Table 1, Supplemental Figure S4a). Furthermore, PCR analysis with primers internal to the *STE2* coding region (Primers P11 and P12—Supplemental Table 1) showed a complete absence of bands. To validate that any phenotypic and chemotropic changes observed are solely due to the deletion of *STE2* and not any off-target genetic defects, a complement strain was constructed by re-introducing the *STE2* gene into the *Fgste2Δ-5* strain (Supplemental Figure S4b). The complemented geneticin-resistant strain was verified by PCR using geneticin-specific and genomic DNA-specific primers to amplify across the junction of the cassette. Sequencing of the genomic DNA showed that the complemented gene was integrated into the native *STE2* locus.

**Figure 4:**
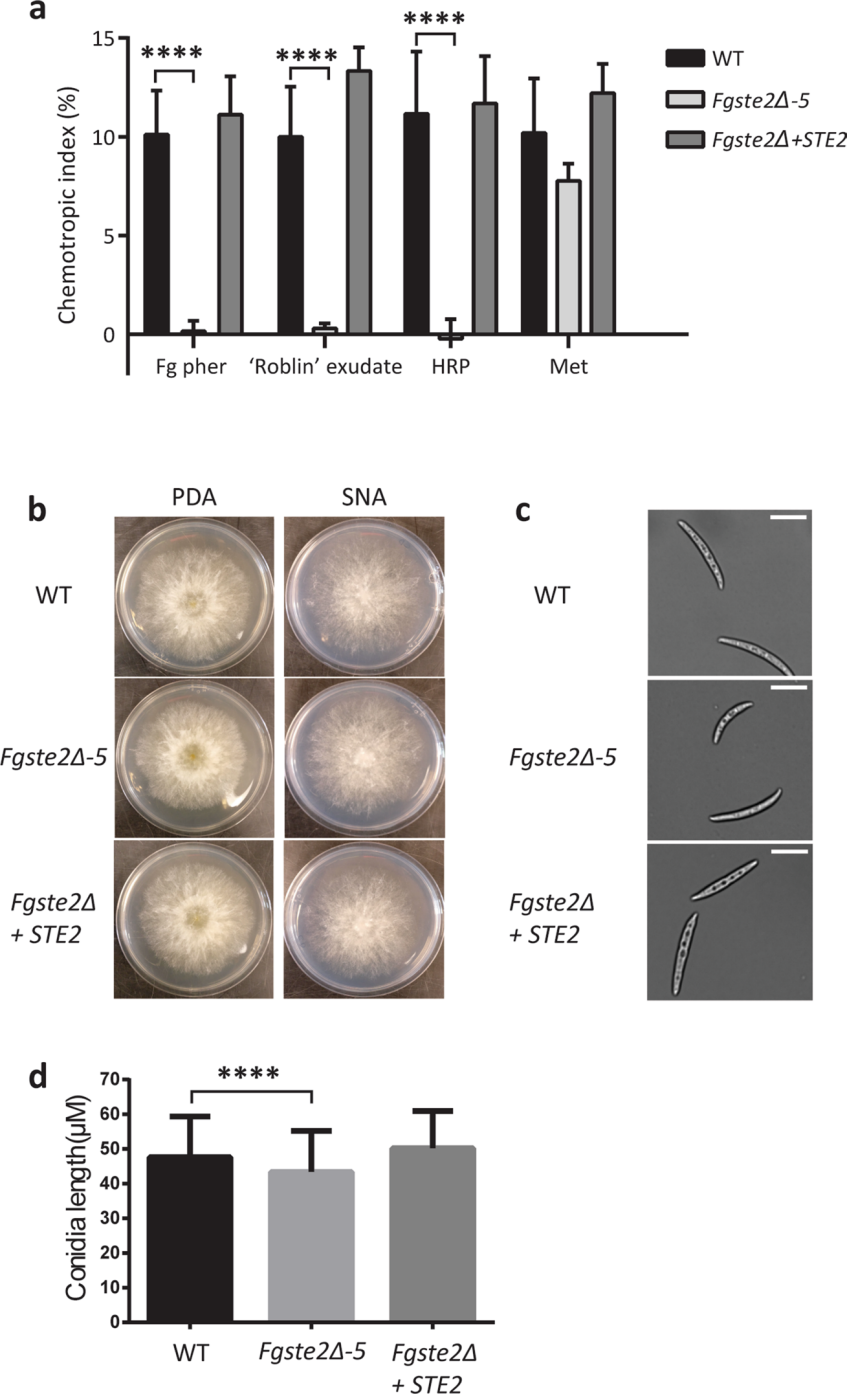
Chemotropism of *F. graminearum* towards α-pheromone and peroxidases is mediated by the Ste2 receptor. (**a**) Directed hyphal growth of wild type, *Fgste2Δ* and *Fgste2Δ* + *STE2* strains of *F. graminearum* towards a gradient of the indicated chemical stimuli (versus water control, ****P < 0.0001). n = 500 hyphae. Data represents the average of at least three replicates. Error bars represent standard deviation. (**b**) Images of wild type and *STE2* mutant strains of *F. graminearum* inoculated on PDA and SNA solid media plates. (**c**) Wild type and *STE2* mutant conidia of *F. graminearum* imaged under 20 × magnification. Scale bar represents 20 µm. The image was captured using cellSens software version 1.12 (https://www.olympus-lifescience.com/en/software/cellsens/). (**d**) Average length of conidia of wild type and *STE2* mutant strains (****P < 0.0001). n = 200 conidia. Error bars represent standard deviation. The figure was compiled using Adobe Illustrator CC 2015 (https://www.adobe.com).

The three *Fgste2Δ* strains and one *Fgste2Δ* + *STE2* strain were assayed against compounds that elicited significant chemotropic responses in wild type *F. graminearum*, specifically *Fg* α-pheromone, ‘Roblin’ exudate, HRP and methionine. A chemotropic index of essentially zero was determined for *Fg* α-pheromone for the *Fgste2Δ-5* mutant, indicating random hyphal growth and an inability to sense the pheromone peptide, confirming that deletion of *FgSTE2* eliminates all chemotropic response to α-pheromone (Fig. 4a). Next, it was observed that the *Fgste2Δ*-5 mutant exhibited no chemotropism towards ‘Roblin’ exudate, signifying the *Fg*Ste2 receptor is responsible for mediating chemotropism towards the ‘Roblin’ exudate (Fig. 4a). The same result was observed upon exposure of the *Fgste2Δ*-5 mutant to a gradient of HRP. Interestingly, however, the *Fgste2Δ-5* mutant exhibited a robust response towards methionine, comparable to that observed in wild type strain, indicating that the response to nutrients is mediated independently of the *Fg*Ste2 receptor. All three *Fgste2Δ* transformants exhibited similar chemotropic responses towards the compounds tested (Supplemental Figure S5). Re-introduction of *STE2* into the *Fgste2Δ-5* mutant restored chemotropic responses towards *Fg* α-pheromone, ‘Roblin’ exudate and HRP in *F. graminearum,* confirming the role of the *Fg*Ste2 receptor in sensing these stimuli (Fig. 4a).

### Deletion of *STE2* has no effect on vegetative growth on solid and liquid media

Vegetative growth of *Fgste2Δ-5* and *Fgste2Δ* + *STE2* strains were assessed on PDA and SNA plates (Fig. 4b). On both media, the growth, colony color, and morphology of *Fgste2Δ-5* was comparable to wild type *F. graminearum*. Additionally, growth of all three *Fgste2Δ* mutants and *Fgste2Δ* + *STE2* in liquid CMC and PDB media was found to be comparable to wild type. Interestingly, conidia of *Fgste2Δ-5* mutants are significantly shorter than those of wild type (Fig. 4c,d).

### Deletion of *STE2* leads to decreased virulence on wheat coleoptiles

**Figure 5:**
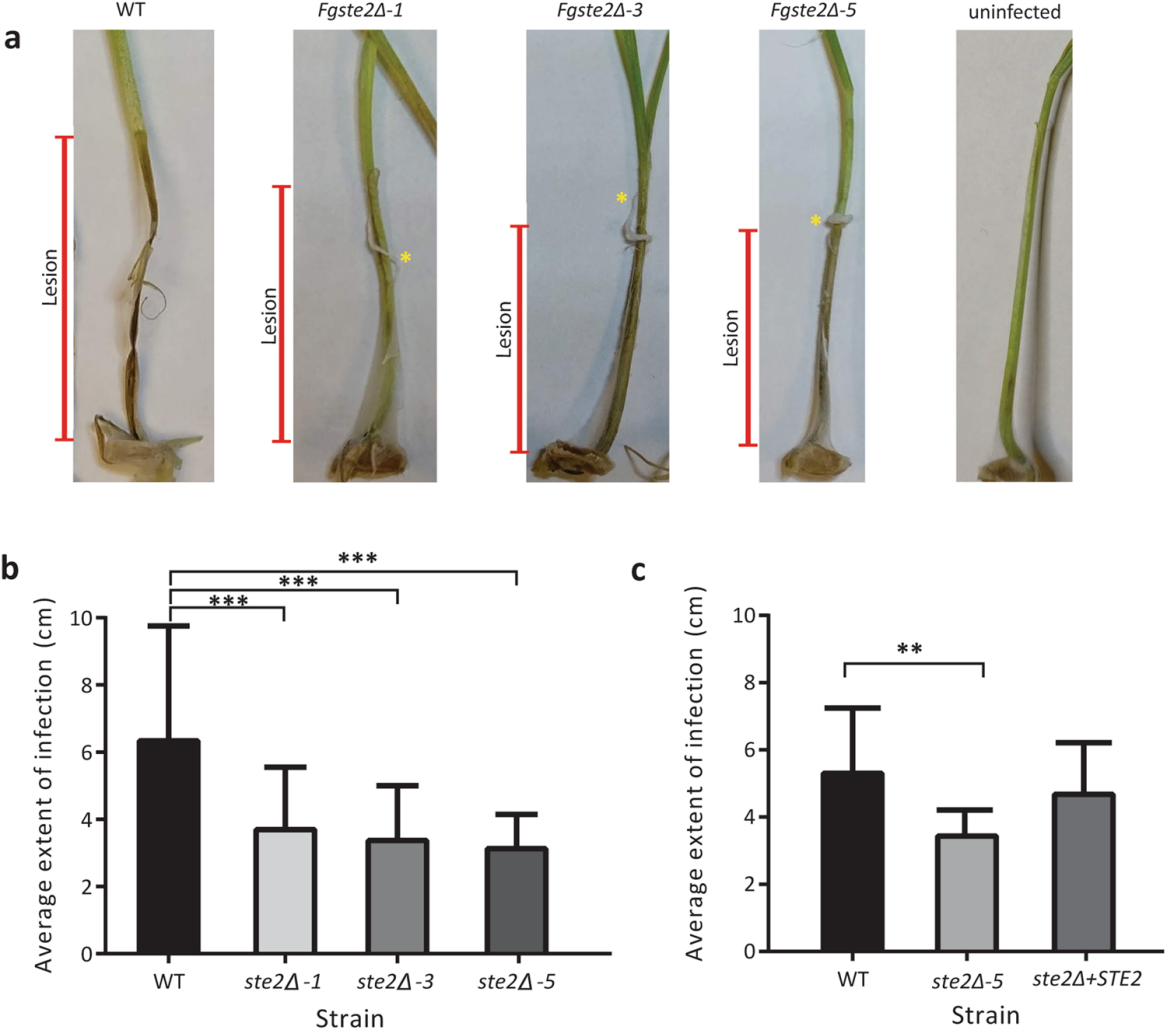
Deletion of *STE2* results in decreased virulence of *F. graminearum* on germinating ‘Roblin’ coleoptiles. (**a**) Representative images of germinating ‘Roblin’ coleoptiles infected by *F. graminearum* strains. The tops of newly germinated coleoptiles were excised using sterile scissors and a cotton thread soaked in a conidial suspension of the strain to be tested was wrapped around the wound site (cotton still visible in some images indicated by an asterisk). Infection of germinating coleoptiles by the indicated strains were imaged and quantified after 10 days of incubation. The length of the stalk that turned brown and necrotic (lesion) is indicated by the red arrows next to each figure and was measured in centimetres using a ruler. (**b**) Average extent of infection of germinating ‘Roblin’ coleoptile stalks infected with the indicated strain of wild type or *Fgste2Δ* mutant strains of *F. graminearum.* Measurements of one representative experiment are shown (compared to wild type strain, ***P < 0.001). Error bars represent standard deviation. n = 15. (**c**) Average extent of infection of germinating ‘Roblin’ coleoptile stalks treated with the indicated strain of wild type or *STE2* mutant of *F. graminearum.* Measurements of one representative experiment are shown (compared to wild type strain, **P < 0.005). Error bars represent standard deviation. n = 12. Graphs were plotted using Graphpad Prism version 6.01 (https://www.graphpad.com). The figure was compiled and labelled using Adobe Illustrator CC 2015 (https://www.adobe.com).

To assess whether *Fg*Ste2 plays a role in pathogenicity, the three *Fgste2Δ* mutants and the *Fgste2Δ* + *STE2* complement strain were assessed in the coleoptile infection assay and compared to wild type *F. graminearum*. The coleoptile assay was selected as a pathosystem to study infection of wheat by *F. graminearum* as it represents a very fast and reliable method, that yields simple and easily quantifiable results. Past studies have shown this coleoptile assay to yield results comparable to wheat head infection assays^36–38^, validating the effectiveness of this assay as a means to assess infection of wheat by *F. graminearum*. Pathogenicity was quantified by measuring the extent of the lesion formed on the ‘Roblin’ coleoptile stalk after 10 days of incubation with *F. graminearum* conidia (Fig. 5a). Inoculation of coleoptiles with wild type *F. graminearum* resulted in dense mycelial growth originating from the wound site, as well as formation of a 5.3 ± 1.9 mm lesion on the stalk. Conversely, all three *Fgste2Δ* strains showed a significant decrease in the extent of infection of the coleoptile (Fig. 5b). As expected, reintroduction of a wild type copy of *STE2* restored pathogenicity of *F. graminearum* (Fig. 5c).

### Exposure of *F. graminearum* to peroxidase leads to CWI MAPK signaling

**Figure 6:**
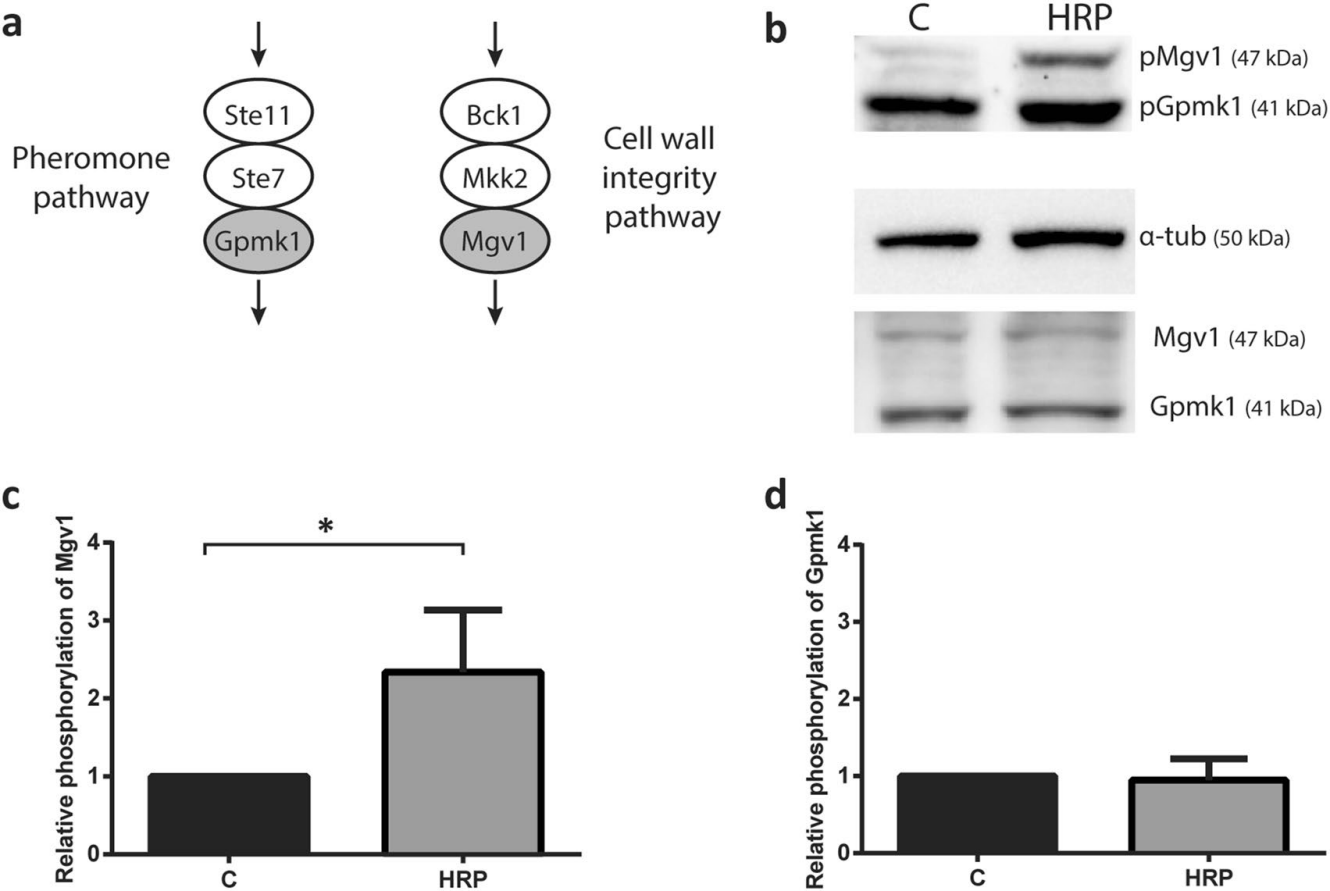
Cell wall integrity MAPK pathway is activated in response to HRP stimulation of *F. graminearum*. (**a**) Elements of MAPK cascade in cell wall integrity and pheromone signaling pathways in *F. graminearum*. MAPKs of interest to this study in each pathway are shaded in grey. Schematic of MAPK was drawn using Adobe Illustrator CC 2015 (https://www.adobe.com). (**b**) Representative immunoblot of MAPKs in the CWI and pheromone signaling pathways, *Fg*Mgv1 and *Fg*Gpmk1. Phospho- and total MAPK for both were probed for in an untreated control (C) and HRP-induced (HRP) condition in wild type *F. graminearum*. For normalization of quantification, α-tubulin was used. Molecular weights of detected proteins are indicated on the blot. Images were cropped using ImageJ (https://imagej.nih.gov/ij/). (**c,d**) Quantification analysis was performed using ImageJ software. The intensity of pMgv1 and pGpmk1 bands were normalized to tubulin, and the ratio of intensities of induced compared to uninduced samples were determined (compared to uninduced sample, *P < 0.05). Data represents the average of three independent experiments. Error bars represent standard deviation. Graphs were plotted using Graphpad Prism version 6.01 (https://www.graphpad.com). The figure was compiled using Adobe Illustrator CC 2015 (https://www.adobe.com).

As Ste2-mediated stimuli has been shown to be transduced through MAPK signaling pathways, immunoblotting was used to monitor the phosphorylation of *Fg*Gpmk1 of the invasive growth MAPK pathway and *Fg*Mgv1 of the CWI MAPK pathway upon exposure to HRP (Fig. 6a). Untreated wild type *F. graminearum* exhibited a basal level of both phosphorylated Mgv1 and Gpmk1 (Fig. 6b). Exposure to HRP for 1 h resulted in a 2.5-fold increase in phosphorylation of *Fg*Mgv1 compared to the control (Fig. 6c), whereas no significant difference in *Fg*Gpmk1 phosphorylation was observed (Fig. 6d).

**Figure 7:**
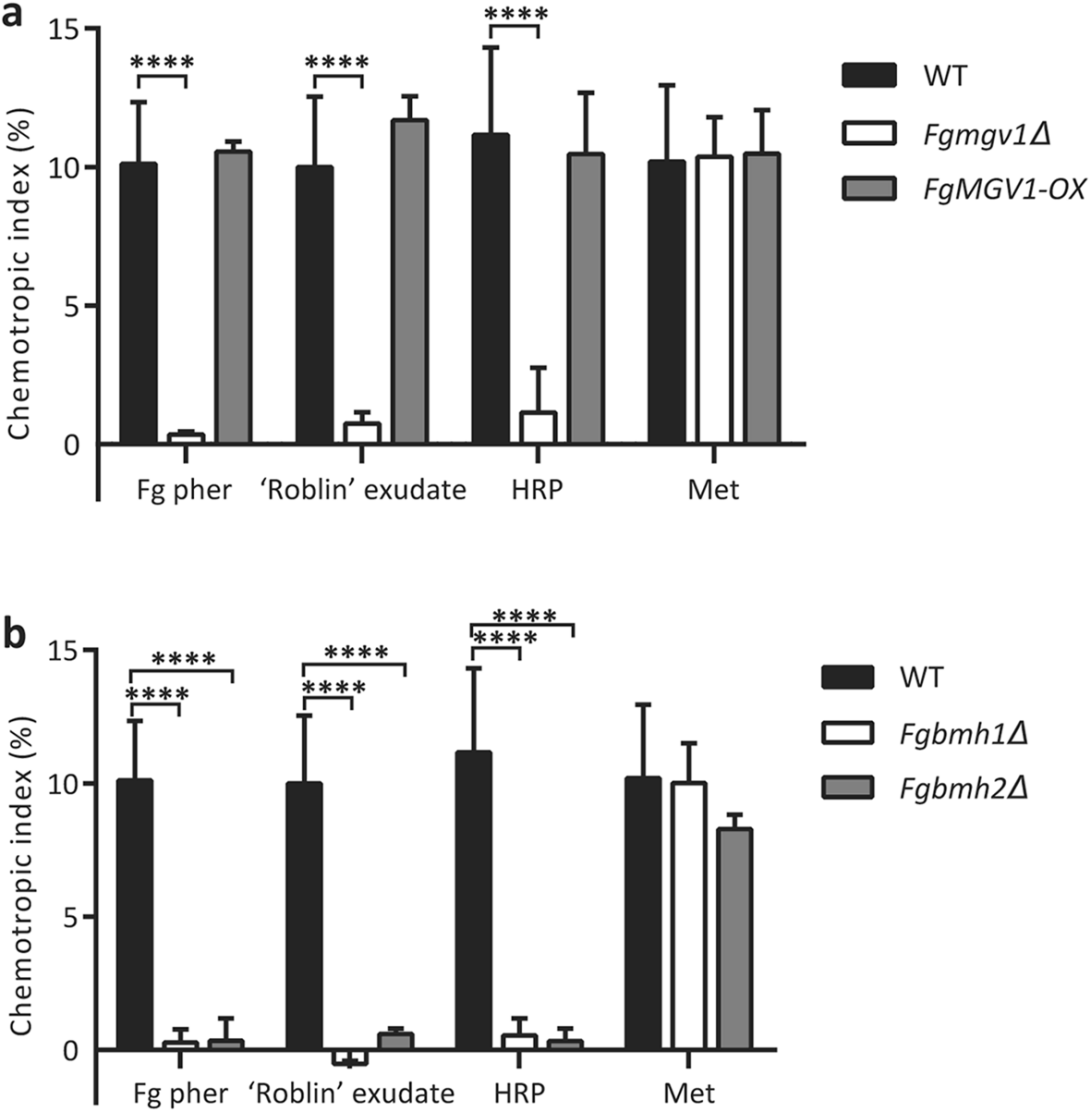
Elements of and/or associated with the CWI MAPK pathway are involved in mediating chemotropic growth of *F. graminearum* towards HRP. Directed hyphal growth of wild type, (**a**) *FgMGV1* and (**b**) *Fgbmh1Δ* and *Fgbmh2Δ* strains of *F. graminearum* towards a gradient of the indicated chemical stimuli (versus water control, ****P < 0.0001). n = 500 hyphae. Data represents the average of at least three replicates. Error bars represent standard deviation. Graphs were plotted using Graphpad Prism version 6.01 (https://www.graphpad.com). Figure was compiled using Adobe Illustrator CC 2015 (https://www.adobe.com).

To further understand the signal transduction pathway initiated by *Fg*Ste2 activation, chemotropism was assessed for *F. graminearum* lacking selected genes involved in, and/or associated with, the CWI MAPK pathway. A deletion mutant of the MAPK in the CWI pathway, *Fgmgv1Δ*^39^ (kindly provided by Dr. Rajagopal Subramaniam), lacked all chemotropic response towards *Fg* α-pheromone (Fig. 7a), signifying that this pathway is involved in relaying the signal from pheromone-activated *Fg*Ste2. The *Fgmgv1Δ* mutant was also unable to sense and grow towards ‘Roblin’ exudate, or HRP, further supporting the fact that *F. graminearum* requires this pathway for sensing and mediating chemotropism towards the host. However, the *Fgmgv1Δ* strain retained the ability to sense and grow towards methionine, reiterating that the pathway responsible for chemotropism towards the host is separate from that involved in nutrient response.

A second *MGV1* mutant strain of *F. graminearum* with constitutively overexpressed *MGV1* (*FgMGV1*-*OX*) was generated and characterized (Supplemental Figure S6a, c). Despite the expression of *FgMGV1* in this mutant being four times higher than wild type (Supplemental Figure S6b), the chemotropic responses observed in *FgMGV1*-*OX* towards *Fg* α-pheromone, ‘Roblin’ exudate, HRP, and methionine did not differ significantly from those observed in wild type *F. graminearum* (Fig. 7a).

Bmh1 and Bmh2 are 14-3-3 adaptor proteins known to associate with the CWI MAPK signaling pathways in *S. cerevisiae*^40^. Orthologues of these proteins in filamentous fungi have been implicated in a variety of processes, including cell cycle progression and cell growth^41,42^, germ tube development and growth^43^, and even repression of secondary metabolite production^44,45^. Single deletion mutants of these orthologous *F. graminearum*^46^ proteins, *Fgbmh1Δ* and *Fgbmh2Δ*, were tested in the chemotropism plate assay (Fig. 7b). When exposed to *Fg* α-pheromone, ‘Roblin’ exudate or HRP, no chemotropic responses were observed in either *Fgbmh1Δ* or *Fgbmh2Δ* (Fig. 7b). Lastly, both *Fgbmh1Δ* and *Fgbmh2Δ* retained robust, wild type-like responses to methionine.

## Discussion

Since the first report of gene disruption in *F. graminearum* in 1995^47^, several studies have been implemented to investigate the role of candidate virulence factors and other fungal genes involved in the interaction between *F. graminearum* and its cereal hosts. However, knowledge about the fungal cell surface receptors that sense and mediate chemotropic growth of *F. graminearum* has been lacking. The primary objective of this research was to understand the mechanisms underlying host sensing and chemotropism by *F. graminearum*.

Many of the nutrients tested in our study have previously been assessed in the context of growth stimulation and mycotoxin induction in *F. graminearum,* but not chemotropism. Unexpectedly, *F. graminearum* exhibited only weak and variable chemotropic responses towards the carbon sources tested, in contrast to the strong response that was observed in *F. oxysporum* toward glucose^5^. While glucose and galactose have been shown to stimulate growth in *F. graminearum*, they are not important for pathogenic traits such as mycotoxin synthesis^48^, consistent with the findings reported here that they do not serve as chemotropic stimuli. Among nitrogen-containing compounds, methionine induced the most robust chemotropic response, as well as extensive hyphal growth and branching in wild type *F. graminearum*. Methionine uptake has been demonstrated to be involved in the induction of trichothecene mycotoxins^49^. Although *F. oxysporum* did not respond to methionine^5^, other filamentous fungi such as *Achlya bisexualis*^50,51^ and *Achlya ambisexualis*^52^ exhibit methionine-driven chemotropism with similar hyphal branching patterns as observed for *F. graminearum*. In agreement with our observations, aspartate and glutamate have previously been shown to induce growth in *F. graminearum*^49^. Interestingly, previous work also identified wheat anthers^53^, and specifically choline and betaine, as selectively stimulating *F. graminearum* conidial growth in a plate assay^54–57^. The lack of chemotropic response towards betaine herein, suggest that while it is likely a growth stimulant of *F. graminearum*, it does not induce chemotropism.

For a long time, research on fungal G-protein-coupled receptors was largely limited to the Ste2p receptor of *S. cerevisiae,* which was used as a model system to study GPCR signaling and mating in yeast. Over the past two decades, pheromone receptors in other fungi, including filamentous fungi like *F. graminearum*^27^, were identified and their roles in mating or sexual reproduction characterized. Pheromone-induced Ste2- and Ste3-mediated chemotropism has been observed in *S. cerevisiae* and *Neurospora crassa*^58^, respectively, and both of these organisms rely on this process for mating and sexual reproduction. More recently, Ste2- and Ste3-mediated chemotropism towards α- and a-pheromone was demonstrated in *F. oxysporum*^5,25^. Furthermore, this pair of receptors has been shown to be involved in the regulation of autocrine pheromone signaling and conidial germination in *F. oxysporum*^25^. While the most obvious role for Ste2 in mating has been extensively studied in heterothallic organisms, its relevance in homothallic fungi such as *F. graminearum* remains enigmatic. Despite not needing a partner for sexual reproduction, *F. graminearum* can only undergo sexual reproduction when both Ste2 and Ste3 are co-expressed; deletion of either *MAT* gene results in an obligate heterothallic strain that can be outcrossed^59^. Our results highlight that even though *F. graminearum* does not need to mate to reproduce, the Ste2 receptor-driven chemotropism mechanism observed in other fungi is maintained in this species.

In addition to an α-pheromone response, *Fg*Ste2 also contributed to the detection of and mediation of chemotropism towards the activity of peroxidases from both wheat and horseradish. This demonstrates the ability of *Fg*Ste2 to recognize multiple ligands and reveals the conserved role of Ste2 in a second *Fusarium* species in host-sensing. Specifically, *Fg*Ste2 responds to the catalytic product of a class III peroxidase secreted from the wheat head. Production of reactive oxygen species (ROS) and secreted peroxidases is a universally utilized strategy by plants to defend themselves against pathogens^60–62^. Indeed, wheat infected with *F. graminearum* ^63,64^ and other pathogens such as *Puccinia triticina*^65^ show increased expression of peroxidases up to 48 h after infection. Additionally, a previous study from our group^33^ showed a significant upregulation of three of the four wheat peroxidases identified in the ‘Roblin’ exudate in wheat infected with *F. graminearum*. Despite the higher amounts of peroxidase secreted by *F. graminearum*-infected wheat, the significantly lower extent of infection by the *Fgste2Δ* strains placed directly on the wound site emphasizes the importance of the *Fg*Ste2 receptor in pathogenicity.

*F. graminearum* encodes three MAPK proteins orthologous to those found in *S. cerevisiae*. These are *Fg*Mgv1, *Fg*Gpmk1 and *Fg*Hog1 with ascribed function in cell wall integrity and remodelling^28^, pathogenicity and invasion^29,30^, and osmotic stress response^31^, respectively. All three MAPKs have been implicated in pathogenicity in filamentous fungal pathogens (reviewed by di Pietro et al.^32^). The Ste2-mediated response to HRP was found to be governed by the cell wall integrity pathway, similar to that observed in *F. oxysporum*^5^. Consistent with this, selective deletion of *Fg*Mgv1 from *F. graminearum* completely abolished all chemotropism towards ‘Roblin’ exudate and HRP. Overexpression of *FgMGV1* had no effect on chemotropic growth compared with wild type, likely due to the number of *Fg*Ste2 receptors involved in the detection of the stimuli remaining constant. Interestingly, a recent study by Jiang et al. found that wild type *F. graminearum* exhibits a two and a half-fold increase in phosphorylation of *Fg*Gpmk1 and negligible increase in phosphorylation of Mgv1 when treated with dissected flowering wheat spikelets^66^. The discrepancy between the aforementioned study and the findings presented here is likely related to methodological differences, where dissected tissues may have a loss of enzymatically activate peroxidases compared to the live plant. In the classical *S. cerevisiae* yeast model, stimulation of Ste2p by α-pheromone results in the recruitment and activation of the Fus3/Kss1 pheromone response MAPK signaling cascade^13^. *Sc*Ste2p activation by α-pheromone also leads its associated Gβγ recruiting Rho1, an effector of the CWI pathway, demonstrating evidence in yeast for *Sc*Ste2p-mediated recruitment of the CWI pathway^67^. It should be noted though, that *Sc*Slt2p in *S. cerevisiae* (orthologue of *Fg*Mgv1) can be activated upon exposure to α-pheromone in the absence of its associated MAPKKK (*Sc*Bck1p), suggesting alternate mechanisms of CWI pathway activation in the presence of pheromone^68^. Yet another protein, the transmembrane sensor, *Fg*Sho1, in *F. graminearum* modulates signaling via both the CWI and invasive growth MAPK signaling pathways^69^. Our results show the existence of a similarly complex and intricate cross-communication between receptors and signaling pathways in *F. graminearum*.

Unlike root-colonizing fungi that exhibit chemotropism towards compounds diffusing through the soil, *F. graminearum* conidia are dispersed from overwintering crop debris onto the wheat head through various physical means and are thus already on the wheat head prior to initiation of chemotropism. Based on the findings presented herein, two mechanisms of peroxidase-stimulated chemotropism of *F. graminearum* are proposed. First, *F. graminearum* secretes cell wall degrading enzymes (CWDE) that would stimulate an increase in peroxidase secretion by the wheat to reinforce the cell wall at that site^61,70^. Higher concentrations of peroxidase-derived chemoattractant could increase the affinity or probability of *F. graminearum* invasion. Second, these aforementioned sites of increased peroxidase secretion would result in the release or diffusion of peroxidase-derived product and attract more distal conidia to invade at these same sites. This is supported by our finding that conidia of *Fgste2Δ* strain placed directly on a wound site displayed a significantly lower extent of infection when compared to the wild type strain. Elements involved in relaying the peroxidase-induced stimulus from *Fg*Ste2 have also been implicated in pathogenicity of wheat; deletion of *Fg*Mgv1^28^ and *Fg*Bmh2^46^ result in a significant reduction of pathogenicity. While *Fg*Bmh1 has been reported to be dispensible for *F. graminearum* infection of wheat^46^, it appears to be involved in mediating chemotropic response towards the wheat peroxidase-derived product. *Fg*Bmh1 and *Fg*Bmh2 have been shown to be involved in sensing nitrogen-containing compounds ammonium nitrate and sodium nitrate^46^, however, deletion of either protein does not affect the response of *F. graminearum* towards methionine, reiterating that methionine uptake occurs through a different mechanism.

Conventional methods of controlling *F. graminearum* include application of fungicides and use of more resistant wheat cultivars^71^. More recently, biological control agents^72,73^,small RNA interference^74,75^ and generation of cultivars overexpressing genes conferring *F. graminearum* resistance^76–78^ have emerged as potential methods of managing *F. graminearum*. In any case, existing disease management methods have their limitations^71,79^ and increasing fungicide resistance, limited FHB-resistant wheat cultivars and changing climate conditions are confounding factors in keeping FHB under control. Understanding the mechanism of infection by *F. graminearum* is essential and will potentially result in more specific targets for fungal inhibition to reduce the devastating consequences of this fungal disease.

## Materials and methods

### Fungal strains, culture conditions and maintenance

Fungal strains used in this study are listed in Table 1. Macroconidia from all strains were obtained through cultures in liquid carboxymethylcellulose (CMC) medium^80^ at 28 °C with shaking at 170 rpm in the dark. Routine maintenance of strains was done on Potato Dextrose Agar (PDA) plates. Plugs of *F. graminearum* strains grown on SNA^81^ or conidial suspensions were stored long-term at − 80 °C in 15% glycerol. For macroconidia harvest, the liquid CMC cultures were filtered through four layers of sterilized cheesecloth and the filtrate was centrifuged at 3,400 g for 10 min at 4 °C. The macroconidia were washed in sterile water twice, resuspended in 1–3 mL of sterile water, and quantified with a hemocytometer.

**Table 1:** List of *F. graminearum* strains used in this study.

**Strain**	**Genotype**	**Gene function**	**References**
GZ-3639	Wild type		Dr. Susan McCormick (USDA)
*Fgste2*Δ	*STE2::HPH*	GPCR	This study
*Fgste2*Δ + *STE2*	*STE2::HPH;STE2:GEN*	GPCR	This study
*Fgmgv1Δ*	*MGV1::HPH*	MAPK	Rampitsch et al.^39^
*FgMGV1-OX*	*pGpdA::mgv1*	MAPK	This study
*Fgbmh1Δ*	*BMH1::HPH*	14-3-3	Brauer et al.^46^
*Fgbmh2Δ*	*BMH2::HPH*	14-3-3	Brauer et al.^46^

### Wheat growth conditions

Wheat cultivars ‘Roblin’, ‘Sumai3’ and ‘Wuhan’ were grown in an AC-60 growth chamber (Enconair) at the greenhouse facility in the Dept. of Biology at Queen’s University. Wheat seeds were kindly provided by Dr. Thérèse Ouellet (AAFC, Ottawa, Canada). Growth light and temperature conditions were 20 °C day, 16 °C night, with a 16 h photoperiod (750 µmol photons/m^2^ × s). Soil was made of 1:1:1 topsoil, sand, Pro-Mix. Fertilizer solution “20-20-20” at a concentration of 2 g/L was administered weekly.

### Quantitative chemotropism plate assay

Chemotropism assays were performed as described previously^5^, with minor modifications. Briefly, fresh *F. graminearum* macroconidia were mixed with 0.5% (*w/v*) water agar to a final concentration of 2.5 × 10^5^ spores per mL and plated in a Petri dish. A scoring line was drawn down the middle of the plate and two wells were made 5 mm away and parallel to the scoring line. Equal volumes (50 µL) of sterile water and test compound were pipetted into the control well and test well, respectively. Tested compounds were: 50% (*v/v*) methanol (MeOH), ammonium sulfate ((NH_4_)_2_SO_4_), glucose (Gluc), glycerol (Glyc), galactose (Gal), all at 50 mM; methionine (Met), sodium aspartate (Asp), sodium glutamate (Glu), all at 295 mM; and 0.1 M betaine. Chemotropic response of *F. graminearum* towards wheat was tested for each of three cultivars. To measure chemotropism towards wheat, the flowering wheat head still attached to the live plant was placed directly into the test well containing sterile water (as shown in Supplemental Figure S2a). Plates were incubated for approximately 14 h at 22 °C in the dark. The number of germinating hyphae growing towards the test (N_test_) or control compound (N_cont_) were counted under the Nikon SMZ1000 microscope and a chemotropic index was calculated as C.I. = $\frac{Ntest-Ncontrol}{Ntest+Ncontrol}\times 100\%$. While only hyphae with angles of approximately 45° or less with respect to the direction of the gradient of test or control compounds were included in the count, no strict criteria for inclusion of hyphae based on length was used. For each compound, a minimum of 500 macroconidia per plate were counted. All experiments were repeated at least three times. Statistical analyses were conducted using Student’s *t*-test and one-way ANOVA on GraphPad Prism version 6.

Commercially available horseradish peroxidase (HRP) was assayed at a concentration of 4 µM. To study the chemoattractive nature of HRP, the enzyme was inhibited by salicylhydroxamic acid (60 mM) (SHAM) for 5 min, heat-denatured at 95 °C for 10 min or proteolyzed by proteinase K (1 mg/mL) for 30 min at room temperature and then assayed. Synthetic *F. graminearum* (*Fg*) (WCTWKGQPCW) and *S. cerevisiae* (*Sc*) (WHWLQLKPGQPMY) α-pheromone peptides were synthesized. Pheromones were reconstituted in 50% (*v/v*) methanol in water and used in the chemotropism assay at a final concentration of 378 µM (final methanol concentration 2.5%). *Fg* α-pheromone was treated with proteinase K solution (1 mg/mL) for 30 min. The reaction was stopped with 1 mM phenylmethylsulfonyl fluoride (PMSF) and tested in the chemotropism assay.

For hyphal length and angle measurements, light microscopy images of chemotropism plates containing HRP as the test compound were taken on an Olympus SZX10 microscope fitted with a DP27 camera. Hyphal lengths and angles, with respect to the HRP gradient, of at least 300 germinating conidia were measured using ImageJ^82^. The experiment was performed twice. Statistical analysis was conducted using Student’s *t*-test.

### ‘Roblin’ exudate production and identification of associated wheat peroxidases

Two flowering ‘Roblin’ wheat heads still attached to the plant were submerged in 25 mL of sterile water and incubated for 48 h at room temperature. Exudate was concentrated either 200- or 300-fold using an Amicon Ultra centrifugal filter (Millipore, cutoff 3 kDa) and stored at 4 °C until further usage in chemotropism or peroxidase activity assays and for protein sequencing. The exudates were assayed directly for peroxidase activity in 96-well plates at 22 °C as described previously^83^. The formation of the pyrogallol oxidation product (extinction coefficient, ε_420_, 4,400 M^-1^ cm^-1^) was measured spectrophotometrically at 420 nm.

Concentrated ‘Roblin’ exudate was run on a 10% SDS–polyacrylamide gel and stained with Coomassie blue. The resolved bands corresponding to proteins of molecular weight 34 and 37 kDa were cut out of the gel and sent to Mass Spectrometry Research, SPARC Biocentre, Hospital for Sick Children, where they were subjected to tryptic digestion. The peptide fragments were analyzed by LC–MS/MS and used to identify the proteins present in the corresponding bands. Data was analyzed using the Scaffold 4 software.

### Fungal genomic DNA isolation

Mycelia were collected from two-day-old *F. graminearum* liquid cultures through filtration, and ground into a fine powder in liquid nitrogen. Genomic DNA (gDNA) was then isolated from the ground tissue using the E.Z.N.A Fungal DNA Mini Kit and eluted in sterile water.

### Construction of vectors

Vectors used for generating the various mutant strains were constructed using Uracil-Specific Excision Reagent (USER) technology^84,85^. All cloning, sequencing and screening primers used in this study are listed in Supplementary Table 1.

To construct the vector for generating the *STE2* deletion mutant (*Fgste2Δ*), flanking regions of the *STE2* gene, which constitute the homologous recombination sequences (HRS) were amplified by polymerase chain reaction (PCR) with primers P1-P4 from wild type *F. graminearum* gDNA using Pfu Cx Turbo Hotstart polymerase (Agilent Technologies). The amplified fragments were cloned into the pRF-HU2 vector^85^ flanking a *hygromycin B phosphotransferase* (*HPH*) gene using USER enzyme mix (NEB). Correct orientation of the inserts in the plasmid was confirmed through PCR and DNA sequencing (P5-P10).

The vector used to generate the *STE2* complement (*Fgste2Δ* + *STE2*) was constructed by amplifying the *STE2* gene, with 1 kb upstream and downstream flanking regions, using primers P17-P18 and cloned into the linearized pRF-GU vector upstream of the geneticin-resistance gene, *aminoglycoside 3′phosphotransferase* (*GEN*)^85^. The generated vector was verified by PCR (P11-P12 and P26-P27) and sequenced with primers P19-P25.

A similar method was employed in generating the *MGV1* over-expression vector using the pRF-HU2E vector which is designed for *in locus* overexpression driven by the *Aspergillus nidulans* GAPDH promoter^85^, was used. In this case, primers P28-P31 were used to generate the expression cassette in pRF-HU2E.

### *Agrobacterium tumefaciens*-mediated transformation (ATMT)

Vectors were transformed into *Agrobacterium tumefaciens* strain LBA4404 by electroporation, and transformants were confirmed by PCR. The reagents and protocol required for ATMT were described by Frandsen^86^.

Correct integration of the knockout cassette and replacement of *STE2* with *HPH* was confirmed by PCR amplification, across the upstream junction formed between the genomic DNA and knockout cassette following homologous recombination with primers P13-P16. Insertion of the complementation cassette was confirmed by PCR with primers P11-P12 and P26-P27. The site of integration of the complementation cassette was confirmed in a similar manner to that of the knockout strain using primers P26-P16 and sequencing of the region of cassette integration into the genomic DNA.

Correct insertion of the *MGV1* overexpression cassette into *F. graminearum* was corroborated using the primers P32-P37 and the *MGV1* sequence was confirmed through sequencing at the Genome Quebec service lab (Montréal, Québec, Canada). This was followed by confirmation of single copy *in locus* insertion of the *MGV1* transgene by quantitative PCR (qPCR). gDNA was isolated from five-day-old *F. graminearum* mycelium culture on PDA plate using the DNeasy Plant Mini Kit (QIAgen). qPCR was employed on the gDNA of the *MGV1* overexpression strains (*FgMGV1-OX*) and the wild type strain to determine the copy number of *MGV1* (amplified with primers P38-P39) and the housekeeping gene *ß -TUBULIN* (primers P40-P41) which served as a control. qPCR was carried out using PerfeCTa SYBR Green SuperMix Low ROX (Quantabio) on a Quant Studio 6 Flex Real-Time PCR System (Applied Biosystems) as described previously^87^. Copy number estimation was calculated against the standard curve, which was generated using the *MGV1* and *ß-TUBULIN* genes (previously amplified from the wild type gDNA).

The overexpression of *MGV1* was verified through reverse-transcription qPCR (RT-qPCR). RNA extraction, cDNA synthesis and qPCR were carried out as previously described^87^. The qPCR reaction was performed in 10 µL with PerfeCTa SYBR Green SuperMix Low ROX (Quantabio) and primers P38-P41. Three biological replicates with three technical replicates were included in each reaction along with the negative controls. Standard curve calculations were used to normalize the data to housekeeping genes and to estimate the relative expression of *MGV1* in *FgMGV1-OX* compared to wild type using the QIAgen’s Relative Expression Software Tool (REST) (p < 0.05).

### Coleoptile infection assay

Infection of germinating coleoptiles with the various *STE2* mutants was carried out as previously described^88^. Briefly, 16 ‘Roblin’ seeds per strain to be tested were placed on ½ MS media in 0.7% (*w/v*) agar in water in autoclaved Magenta boxes and stratified overnight at 4 °C in the dark. The Magenta boxes were then placed in the growth chamber and coleoptiles were grown until at least 1 cm in height, where 12–16 seeds germinated per strain. Sterile scissors were used to cut 1 mm off the top of the coleoptile and a cotton soaked with macroconidial suspension (2 × 10^5^ spores per mL) was wrapped around the wound site. The Magenta boxes were placed in the growth chambers to allow symptom development. After ten days, the length of coleoptile stalks infected were measured for each strain. The experiment was performed twice. Statistical analysis was conducted by one-way ANOVA.

### Conidial length quantification

Conidia of wild type and *STE2* mutants of *F. graminearum* were imaged using an Olympus IX83 inverted microscope fitted with a 20 × objective and an Andor Zyla 4.2 Plus camera controlled by the cellSens software. Two hundred conidia were measured per strain and conidial lengths were quantified using Image J. Statistical analysis was conducted by one-way ANOVA.

### Immunoblotting of MAPKs

Approximately 10^5^ spores of wild type *F. graminearum* were inoculated in 20 mL of liquid Potato Dextrose Broth (PDB) and grown for 24 h at 28 °C in the dark. The growing culture was exposed to 0.05 µM HRP or water control for 1 h, and the cells were then lysed as previously described^89^ with some modifications. Briefly, the mycelia were collected by filtering the culture through Whatman filter paper, finely ground in liquid nitrogen and resuspended in 1 mL of protein extraction buffer (1 M NaCl, 50 mM sodium phosphate, pH 8.0, 50 mM NaF, 1 mM PMSF, 0.2% β-mercaptoethanol, protease inhibitor cocktail, and phosphatase inhibitor cocktail). The cell lysate was homogenized by vortexing and then centrifuged at 13,000 × *g*. The supernatant was transferred to a fresh microfuge tube and total protein concentration was quantified with Bradford assay^90^. Twenty micrograms of total protein of each sample were loaded and resolved on a 12% SDS polyacrylamide gel and transferred to a polyvinylidene difluoride (PVDF) membrane by wet electroblotting at 400 mA for 2 h. The membranes were blocked for 1 h in 5% (*w/v*) non-fat dried skim milk in TBST (50 mM Tris, pH 7.5, 150 mM NaCl, 0.05% (*v/v*) Tween 20) at 4 °C. The membranes were subsequently incubated with either anti-p44/42 MAP kinase (1:1,000 dilution, M5670, Millipore Sigma) or anti-phospho p44/42 MAP kinase (1:1,000 dilution, CST #9101, Cell Signaling Technology) primary antibodies. The membranes were then incubated with anti-rabbit IgG secondary antibodies (1:5,000 dilution, 7074S, Cell Signal Technology). Pierce Enhanced Chemiluminescent substrate was added to the membranes and the emitted light was captured on an x-ray film. The same membranes were then re-probed for α-tubulin (1:1000, sc53030, Santa Cruz Biotechnology) as a loading control. Quantification was performed using ImageJ. The experiment was repeated three times with independent sets of samples and analyzed by Student’s *t*-test.

## Supplementary information

Supplementary file1 (PDF 2629 kb)

Supplementary file2 (PDF 174 kb)
