# Supplementary material for: Ste2 receptor-mediated chemotropism of Fusarium graminearum contributes to its pathogenicity against wheat
Source: Sci Rep. 2020 Jul 1;10:10770. doi: 10.1038/s41598-020-67597-z (PMC7329813; doi:10.1038/s41598-020-67597-z)
Supplement: Supplementary file 1 — Supplementary file1 (PDF 2629 kb) [file 41598_2020_67597_MOESM1_ESM.pdf]

Supplemental Figures S1 – S6

Ste2 receptor-mediated chemotropism of *Fusarium graminearum* contributes to its pathogenicity against wheat

Pooja S. Sridhar[1], Daria Trofimova[1], Gopal Subramaniam[2], Dianevys González-Peña Fundora[3], Nora A. Foroud[3], John S. Allingham[1], Michele C. Loewen[1, 4†]

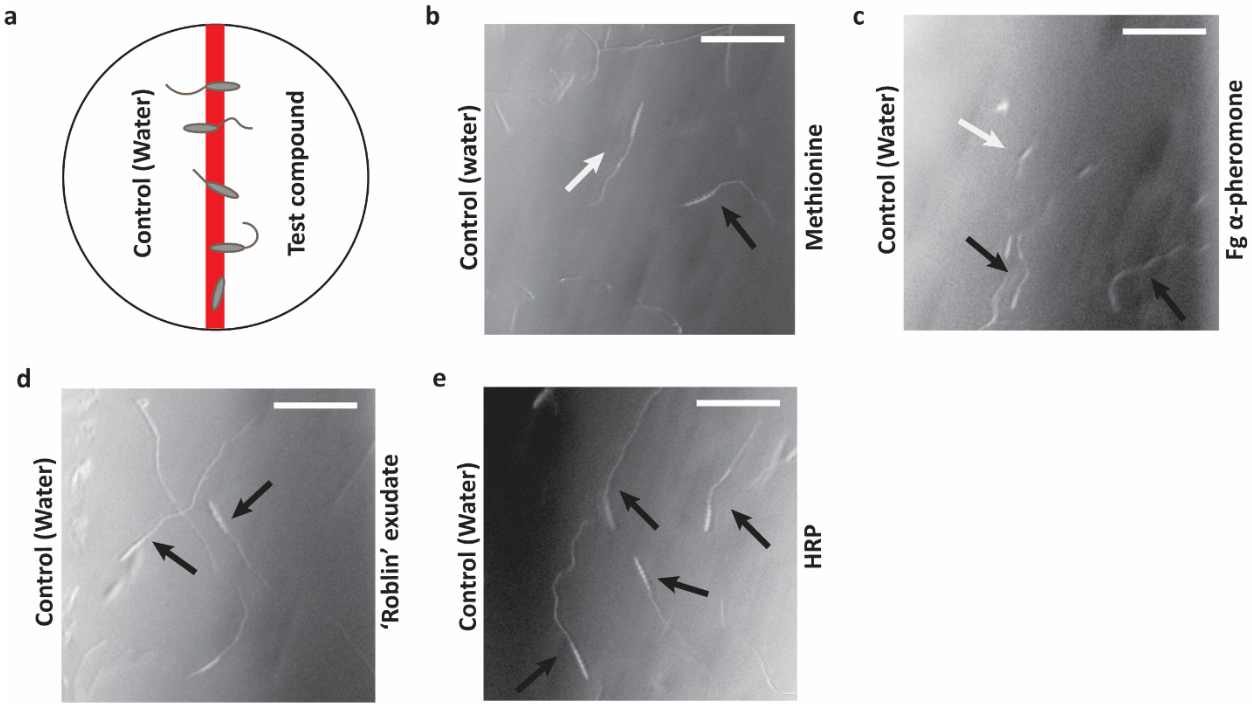

Supplemental Figure S1. Representative images of *F. graminearum* macroconidia in chemotropism plate assays. a. Schematic diagram depicting chemotropism assay, indicating macroconidia on the scoring line with their germinating hyphae. Figure was drawn using Adobe Illustrator CC 2015 (https://www.adobe.com). Representative images taken using a microscope of conidia exposed to gradients of the indicated compounds **c.** methionine, **c.** *Fg* α-pheromone, **d.** 'Roblin' wheat head exudate, **e.** HRP – horse radish peroxidase. Images represent hyphal growth after 14 h of exposure. Image contrast has been adjusted for better visualization of growing hyphae using ImageJ (https://imagej.nih.gov/ij/). White and black arrows represent hyphae growing towards the water control and test compound, respectively. Hyphae not indicated by arrows in the figure were excluded from count. Scale bar represents 0.1 mm. Figure was compiled and labelled using Adobe Illustrator CC 2015 (https://www.adobe.com).

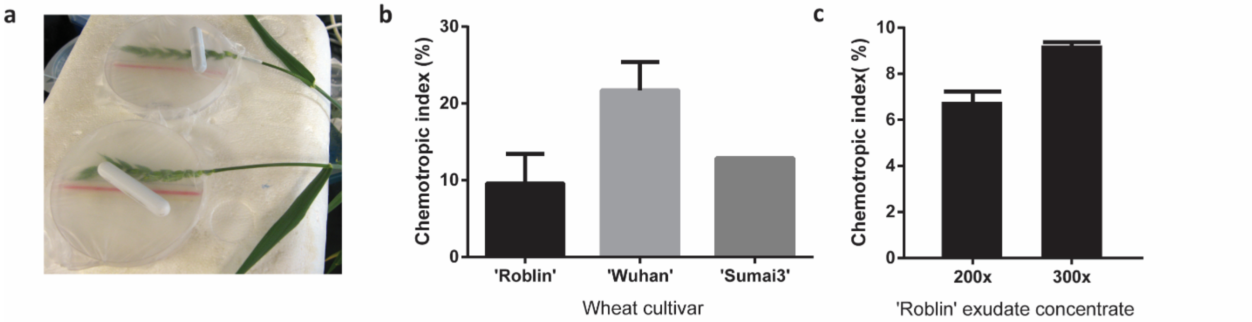

Supplemental Figure S2. Chemotropism of wild type *F. graminearum* towards wheat heads. **a.** Representative image of experimental testing of intact wheat heads still attached to the plant in the chemotropism assay. **b.** Preliminary findings for directed hyphal growth of wild type *F. graminearum* towards wheat heads of the indicated cultivars after 14 h exposure. n = 500 hyphae. Error bars represent standard deviation of 2 repetitions. **c.** Directed hyphal growth of wild type *F. graminearum* towards different concentrations of 'Roblin' exudate after 14 h of exposure. n = 500 hyphae. Error bars represent standard deviation of 2 repetitions. Graphs were plotted using Graphpad Prism version 6.01 (https://www.graphpad.com). Figure was compiled using Adobe Illustrator CC 2015 (https://www.adobe.com).

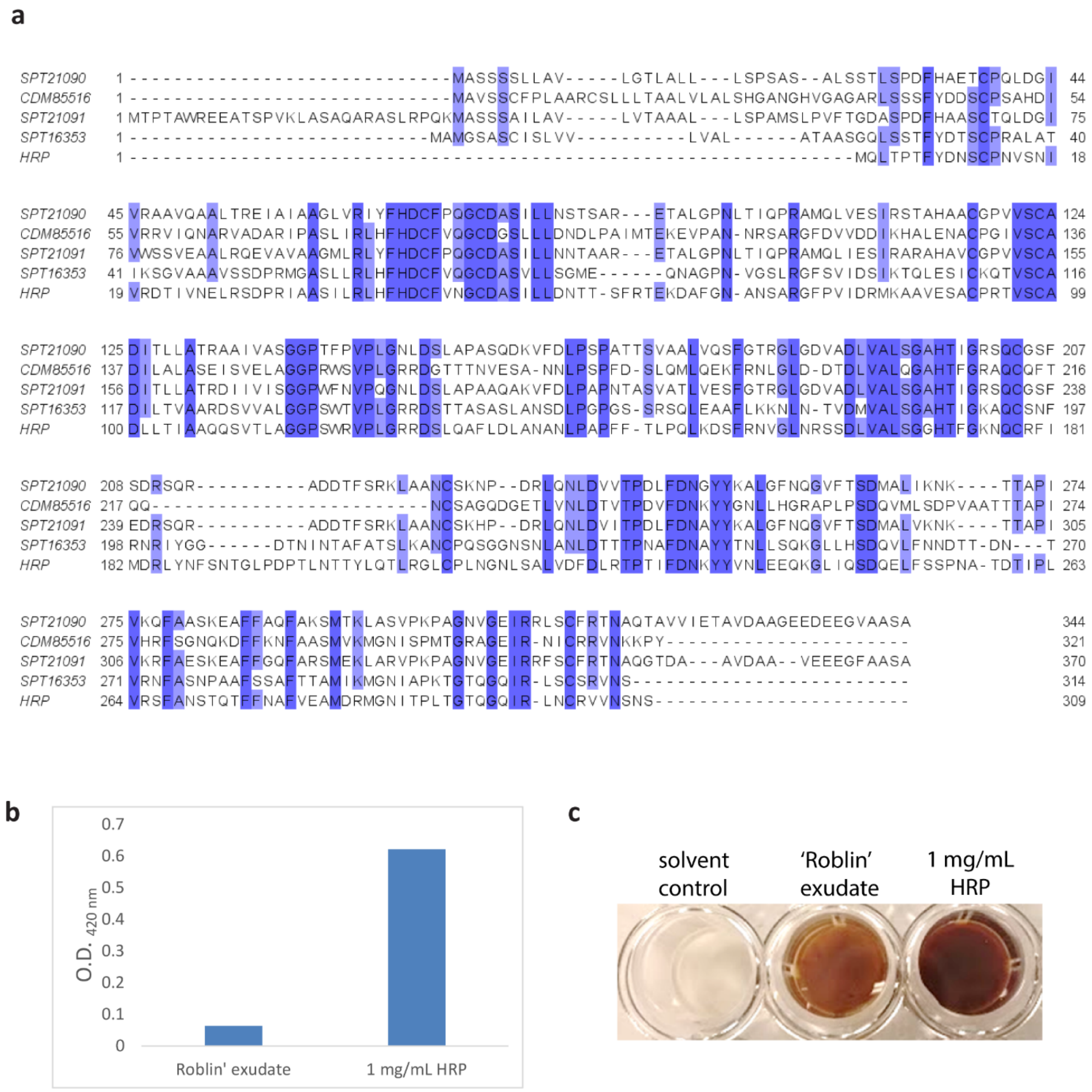

Supplemental Figure S3. Multiple sequence alignment and catalytic activity of plant peroxidases. a. Multiple sequence alignment of 'Roblin' peroxidases identified by mass spectrometry, SPT21090, CDM85516, SPT21091, and SPT16353, with HRP. Residues shaded in dark blue and light blue are highly conserved in all or at least four proteins, respectively. Alignment was performed using Jalview (https://www.jalview.org/). **b.** Peroxidase enzyme activity assay of 'Roblin' exudate with pyrogallol and hydrogen peroxidase substrates. The amount of product formed was measured by spectrophotometry at 420 nm after incubation for 30 sec. Data is representative of one experiment. **c.** Representative image of the peroxidase activity assay depicting reaction carried out at room temperature for 5 minutes. Graph was plotted using Microsoft Excel 365. Figure was compiled using Adobe Illustrator CC 2015 (https://www.adobe.com).

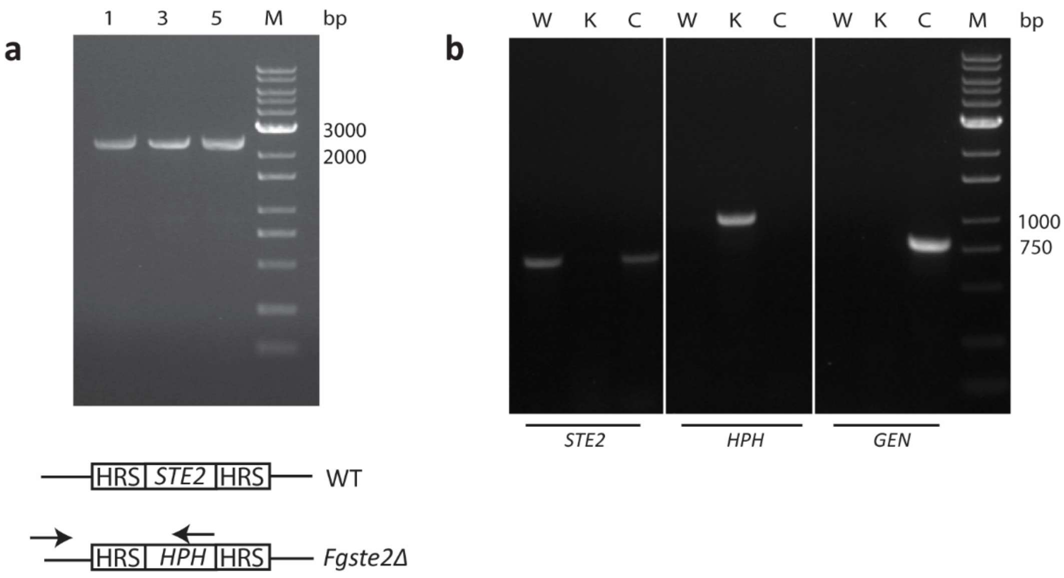

Supplemental Figure S4. Confirmation of various *STE2* mutants of *F. graminearum* by polymerase chain reaction. a. Representative agarose gel of PCR confirmation of localized integration of *STE2* deletion cassette using primers P13 and P16 for the three *Fgste2Δ* mutants, indicated by 1, 3, and 5. Specific primer binding sites are indicated on the schematic diagrams below the agarose gel image. Expected size of the amplified DNA fragment is approximately 2400 bp. **b.** Representative agarose gel summarizing the genotypes of the various *STE2* mutant strains using internal primers for *STE2*, hygromycin B phosphatase (*HPH*) and aminoglycoside 3'-phosphotransferase (*GEN*). W, wild type; K, ste2 knockout (*Fgste2Δ*); C, ste2 complement (*Fgste2Δ+STE2*). Expected sizes of amplified *STE2*, *HPH*, and *GEN* are 657 bp, 983 bp and 784 bp, respectively. Schematic was drawn and figure was compiled and labelled using Adobe Illustrator CC 2015 (https://www.adobe.com).

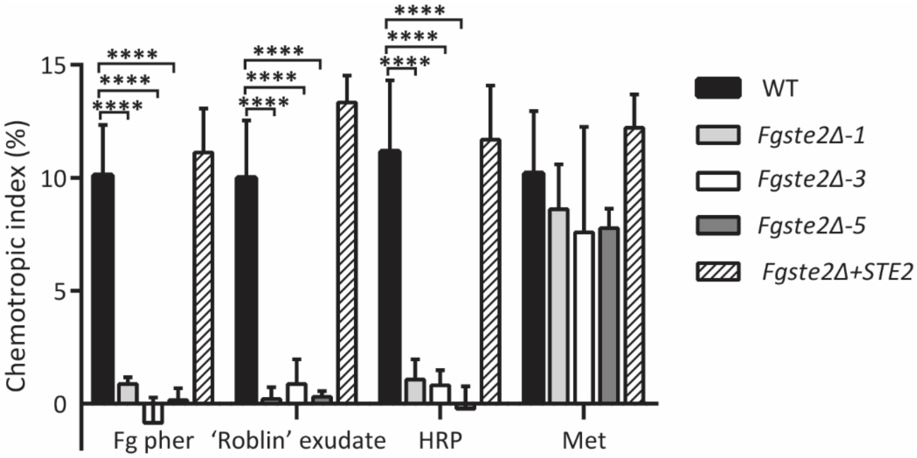

Supplemental Figure S5. Chemotropism of *F. graminearum* towards α-pheromone and peroxidases is mediated by the Ste2 receptor. A. Directed hyphal growth of wild type, *Fgste2Δ* mutant strains and *Fgste2Δ+STE2* strains of *F. graminearum* towards a gradient of the indicated chemical stimuli (versus water control, *****P*<0.0001). n = 500 hyphae. Data represents the average of at least three replicates. Error bars represent standard deviation. Graph was plotted using Graphpad Prism version 6.01 (https://www.graphpad.com).

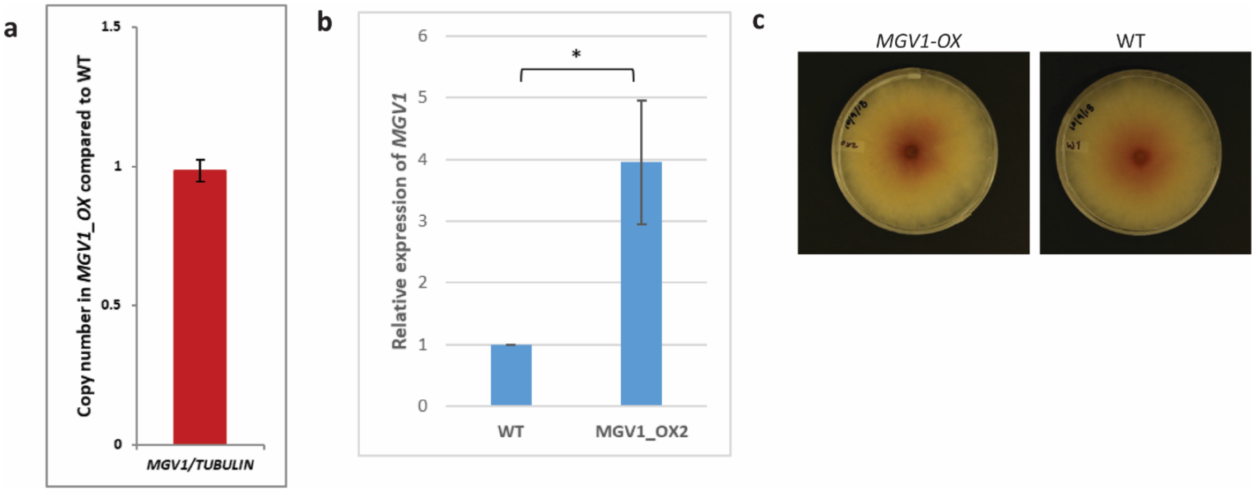

Supplemental Figure S6. Validation and characterization of *MGV1-OX* strain. a. Estimation of number of copies of *FgMGV1* in the *FgMGV1-OX* strain by qPCR normalized to tubulin and relative to wild type *F. graminearum*. **b.** Level of expression of *FgMGV1* in the *FgMGV1-OX* strain relative to wild type was determined by RT-qPCR. Data is representative of three technical replicates. $P<0.05$ **c.** Colony growth of wild type *F. graminearum* and *FgMGV1-OX* on PDA. Graphs were plotted using Microsoft Excel 365. Figure was compiled using Adobe Illustrator CC 2015 (https://www.adobe.com).
