# Supplementary material for: Ste2 receptor-mediated chemotropism of Fusarium graminearum contributes to its pathogenicity against wheat
Source: Sci Rep. 2020 Jul 1;10:10770. doi: 10.1038/s41598-020-67597-z (PMC7329813; doi:10.1038/s41598-020-67597-z)
Supplement: Supplementary file 2 — Supplementary file2 (PDF 174 kb) [file 41598_2020_67597_MOESM2_ESM.pdf]

Supplemental Table 1

Ste2 receptor-mediated chemotropism of *Fusarium graminearum* contributes to its pathogenicity against wheat

Pooja S. Sridhar[1], Daria Trofimova[1], Gopal Subramaniam[2], Dianevys González-Peña Fundora[3], Nora A. Foroud[3], John S. Allingham[1], Michele C. Loewen[1, 4†]

Supplementary Table 1. List of oligonucleotides used this study

Target	F/R	Primer	Sequence (5'→3')	Product size (bp)
Upstream HRS of *STE2*	F	P1	GGGTTTAAU-GTAGTTGGCTCTGGGATTGCG	936
	R	P2	GGACTTAAU-TCGTTGTTGTTGGCGATGGACT	
Downstream HRS of *STE2*	F	P3	GGCATTAAU-GACCTCACGTCTCGATCTACG	723
	R	P4	GGTCTTAAU-TCGATCAAGGCGGACGAGTAA	
Upstream HRS sequencing		P5	GTCGAGGCCTGATCATCGATG	
		P6	GTTGTAAAACGACGGCCAGTGA	
Downstream HRS sequencing		P7	GGAGAATTAAGGGAGTCACGAAGC	
		P8	AGCGTCGAGATCTAGAGGATCC	
HPH sequencing	F	P9	GATCCCGGTCGGCATCTACT	
	R	P10	CGACCCGGTCATACCTTCTTAAGTT	
STE2 gene	F	P11	CTGCCATTGACCAGGTGC	657
	R	P12	AGATGCCGTTGGTCATGATGAG	
HPH gene	F	P13	TGAACTCACCGCGACGTCT	983
	R	P14	GTCGGTTTCCACTATCGGCG	
STE2 knockout cassette localization	F	P15	CGCAGCTCCATTGCTAAGTGG	2372
	R	P16	GTCGGTTTCCACTATCGGCG	
STE2 complementation	F	P17	GGGTTTAAU-GTAGTTGGCTCTGGGATTGCG	2771
	R	P18	GGACTTAAU- TCGATCAAGGCGGACGAGTAA	
GEN sequencing		P19	GCTTGGTGGTCGAATGGG	
		P20	GCATACGCTTGATCCGGCTA	
STE2 complement construct sequencing		P21	CACTCATCAAAAGTCGTATTTCGGAG	
		P22	TGTGGCAAGAAGACACGGG	
		P23	TATGGCTGAACAGGCGATGGA	
		P24	GATTCGAGGTCATTGATCGAAAC	
		P25	AGATTATCTCAAACCGTTCCCG	
GEN gene	F	P26	CAAGATGGATTGCACGCAGG	784
	R	P27	AGAAGAACTCGTCAAGAAGGCG	
Upstream HRS of *MGV1*	F	P28	GGTCTTAAUCTTGGCAAGATTCAGCCCTA	871
	R	P29	GGCATTAAUTTTCCCAAGTTGGTTGGGT	
MGV1 gene	F	P30	GGACTTAAUATGCACCATCACCATCACCATGG CGACCTACAAGGA	1577
	R	P31	GGGTTTAAUTTATCTTCGAGAAGCATCCAGG	
MGV1 upstream HRS sequencing	F	P32	TTTGTCGATCCCGCATCAT	1135
	R	P33	TCTCCTTGCATGCACCATTCCTTG	
MGV1 gene sequencing	F	P34	TTGCGTCAGTCCAACATTTGTTGCCA	2140
	R	P35	CTGCCCTCTCAACCCATTTA	
HPH gene	F	P36	AGCTGCGCCGATGGTTTCTACAA	588

	R	P37	GCGCGTCTGCTGCTCCATACAA	
TUBULIN qPCR	F	P38	GTTCTGGACGTTGCGCATCTG	110
	R	P39	TGATGGCCGCTTCTGACTTCC	
MGV1 qPCR	F	P40	ATGGAGTGTGATTTGGCTGC	85
	R	P41	TGGATGTACTTGAGACCGCA	
